# Efficacy of Neuromuscular Training in Patients of Osteoarthritis Knee via Telerehabilitation

**DOI:** 10.7759/cureus.89407

**Published:** 2025-08-05

**Authors:** Mamta Joon, Ajay Gupta, Suman Badhal, Vijender Anand, Anjana Babu

**Affiliations:** 1 Physical Medicine and Rehabilitation, Vardhman Mahavir Medical College and Safdarjung Hospital, New Delhi, IND; 2 Physical Medicine and Rehabilitation, AIIMS-CAPFIMS Center, New Delhi, IND

**Keywords:** covid-19, exercise, neuromuscular, osteoarthritis knee, telerehabilitation

## Abstract

Introduction: Osteoarthritis (OA) of the knee is a leading cause of pain and disability among adults, resulting in structural and functional compromise of synovial joints. Neuromuscular training (NMT) has demonstrated benefits in managing OA; however, its delivery through telerehabilitation remains underexplored. This study aimed to evaluate the effectiveness of NMT delivered via telerehabilitation in patients with primary knee OA.

Methods: This interventional cohort study included 30 patients diagnosed with primary tibiofemoral OA at the Physical Medicine and Rehabilitation (PMR) outpatient department (OPD) of a tertiary care center. NMT was demonstrated during the initial visit, and patients received a video link for home-based exercises. Telerehabilitation sessions were conducted once weekly for the first two weeks, followed by biweekly sessions for the next 10 weeks for a total duration of 12 weeks. Patients were instructed to perform daily NMT at home for at least 30 minutes. Outcome measures included pain (Visual Analog Scale (VAS)), functional status (Knee Injury and Osteoarthritis Outcome Score (KOOS)), and physical performance (30-second Chair Sit-to-Stand Test (30s-CST), 40-meter Fast-Paced Walk Test (40m-FPWT), and 9-Step Stair-Climb Test (9-Step SCT)).

Results: Significant improvements were observed in all parameters post intervention. Mean changes with 95% CIs were: VAS -40 (CI: -50 to -40), KOOS +21 (CI: 19.62 to 22.38), 30s-CST +3 (CI: 2 to 2), 40m-FPWT -0.29 (CI: 0.12 to 0.20), and 9-step SCT -3.75 (CI: -6.34 to -3.24).

Conclusion: NMT administered via telerehabilitation resulted in significant improvements in pain, functional ability, and physical performance in patients with knee OA. These findings support the implementation of telerehabilitation as an effective and accessible modality for delivering NMT in OA knee management.

## Introduction

Osteoarthritis knee (OA) is a commonly seen chronic degenerative disease that affects a big proportion of the population [1]. OA knee is more commonly seen in people over the age of 50 years causing pain and disability [2,3]. Genetic mutations, obesity, trauma, and local biomechanical factors changing hormones with aging are the major risk factors for OA knee. OA knee constantly impairs patients' activities of daily living (ADLs) and their ability to engage in work, imposing a significant financial burden on society [4].

OA knee is the second most common rheumatological problem with a prevalence of 22% to 39% in India [5]. The estimated prevalence of OA knee in the Delhi population above 18 years of age is 3.28% [6]. Even in the healthy knees, there is increased susceptibility of the medial compartment to degenerative OA presumably as 60%-80% of the entire load passes through the medial tibiofemoral compartment compared to the lateral tibiofemoral compartment throughout gait [7]. This usually leads to or predisposes one to varus malalignment.

Physical therapy, physical exercises and rehabilitation are accompaniments to the available treatment options for OA knee [8]. Appropriate pre- and post-operative physiotherapy can restore quadriceps strength and improve proprioception in patients with post-operative OA knee [9]. Surgery is not recommended for patients with early OA knee. Therefore, finding a non-surgical treatment to effectively relieve the symptoms of OA knee patients is very important.

Exercise training is aimed at improving all physical functions through either the patient's strength or the assisted operation of the therapist or equipment [10]. A systematic review of a randomized trial of therapeutic exercise in OA knee patients showed that exercise could significantly reduce pain, improve physical function and quality of life [11]. In addition, exercise can improve cardiopulmonary function, strengthen muscles, stabilize posture, and improve mental health [12]. Therefore, exercise training is an effective adjunct therapy and plays an important role in the management of patients with OA knee.

Weakness of quadriceps muscles is one of the common findings in OA knee and, therefore, an important component of the exercise program prescribed as a part of conservative management is quadriceps training (QT) [13]. However, strengthening of the quadriceps alone is not sufficient in reducing pain and knee adduction moment (KAM) in patients with OA knee, particularly in patients having varus malalignment [14]. QT primarily improves the strength of muscles (muscle output), instead of aiming at the biomechanical factors which contribute to the medial compartment knee loading [15].

Neuromuscular exercise programs comprise various training such as proprioceptive training, agility training, perturbation training, or functional exercises. For the lower limb, neuromuscular training (NMT) involves multiple joints and muscles that are carried in functional weight bearing positions. It emphasizes not only the quality and efficiency of the movement but also the trunk and lower limb alignment [16]. NMT has been shown to alter the biomechanics and muscle recruitment patterns around the knee joint and in addition, improve functional performance in a population other than individuals experiencing OA knee such as anterior cruciate ligament (ACL) injury or meniscal injury. These exercises have shown benefits in the prevention and rehabilitation of knee injuries within the athletic population [17]. Although there is a lack of research regarding its mechanism, some research has revealed the benefits of neuromuscular exercise in OA knee [18].

COVID-19 has induced governments all over the world to adopt certain rules limiting individual freedom and imposing social distancing (e.g. mandatory quarantine, closing schools, restricting entertainment) to prevent the collapse of healthcare systems at various levels [19]. Although these measures are necessary during this pandemic, they created a barrier for healthcare professionals who are usually in close contact with patients needing low-intensity care, like rehabilitation services. Recently, several recommendations on the management of patients with musculoskeletal pain (e.g. OA, psoriatic arthritis, gout, rheumatoid arthritis, ankylosing spondylitis, backache, neck pain, osteoporosis, etc.) have increasingly highlighted the importance of hands-off approaches to enhance the standard of care and consequently, the standard of life [20]. This time is ripe to utilize the potential of telerehabilitation for patients with musculoskeletal pain. Through telerehabilitation, maintaining social distancing during COVID-19 was possible. At the same time, monitoring patient’s progress and providing them with continuous feedback and supervision could be achieved.

The available evidence shows that telerehabilitation could be comparable to or better than the conventional methods of rehabilitation to reduce pain and improve physical function in musculoskeletal conditions generally. Additionally, telerehabilitation could improve functionality in patients with OA in the knee and non-specific low-back pain in addition to improving quality of life in patients with non-specific low-back pain, OA in the knee and total arthroplasty in the knee and hip [21].

The purpose of this study is to establish a telerehabilitation module (TRM) for NMT in OA knee patients.

Objectives

This study seeks to examine the effect of NMT through telerehabilitation in OA knee using Visual Analog Scale (VAS) and Knee Injury and Osteoarthritis Outcome Score (KOOS) and evaluate physical performance outcomes using 30-second Chair Sit-to-Stand Test (30s-CST), 40-meter Fast-Paced Walk Test (40m-FPWT), 9-Step Stair-Climb and Descent Test (9-Step SCT).

This article was previously presented as a poster at the 2022 American Congress of Rehabilitation Medicine (ACRM) 99th Annual Conference on November 10, 2022.

## Materials and methods

This interventional cohort study was conducted in the Department of Physical Medicine and Rehabilitation (PMR) at a tertiary care hospital from February 2021 to June 2022. After getting the clearance of the Institutional Ethics Committee (IEC/VMMC/SJH/Thesis/2020-11/CC-269) and the Institutional Review Board, the study was registered at Clinical Trials Registry-India (CTRI registration number: CTRI/2021/02/031103). Subjects were included in the study after obtaining written informed consent and satisfying the inclusion and exclusion criterion. Patients of either gender diagnosed with primary OA knee of tibia-femoral joint as defined by Americana College of Rheumatology (ACR) clinical and radiographic criteria with knee pain over the past week ≥ 25 on a 100mm VAS with radiological grade l, ll, lll of knee OA according to Kellgren-Lawrence (KL) classification and having access to smartphones/computer having internet connectivity were included. Google Meet was used as the digital platform for telerehabilitation. It is a free-of-cost platform that needs a Google ID to log in. Exclusion criteria included knee surgery or knee intra-articular treatment within the past six months, current or past (within four weeks) oral corticosteroid use, systemic arthritic conditions, history of hip or knee joint replacement or tibial osteotomy surgery, any other condition affecting lower limb function, participation in any structured exercise program within the past six months, unable to ambulate without a gait aid, uncontrolled diabetes mellitus, known history of coronary artery disease (CAD)/chronic obstructive pulmonary disease (COPD) likely to impact functional evaluation.

Sample size

The study by Rashid et al. observed that the median and interquartile range of Western Ontario and McMaster Universities Osteoarthritis Index (WOMAC) in NMT was 9.37 (3.12) [22]. Takaing this value as a reference, the minimum required sample size estimated to be within 10% and 5% level of significance was 24 patients. To reduce the margin of error, the total sample size taken was 30.

**Figure 1:**
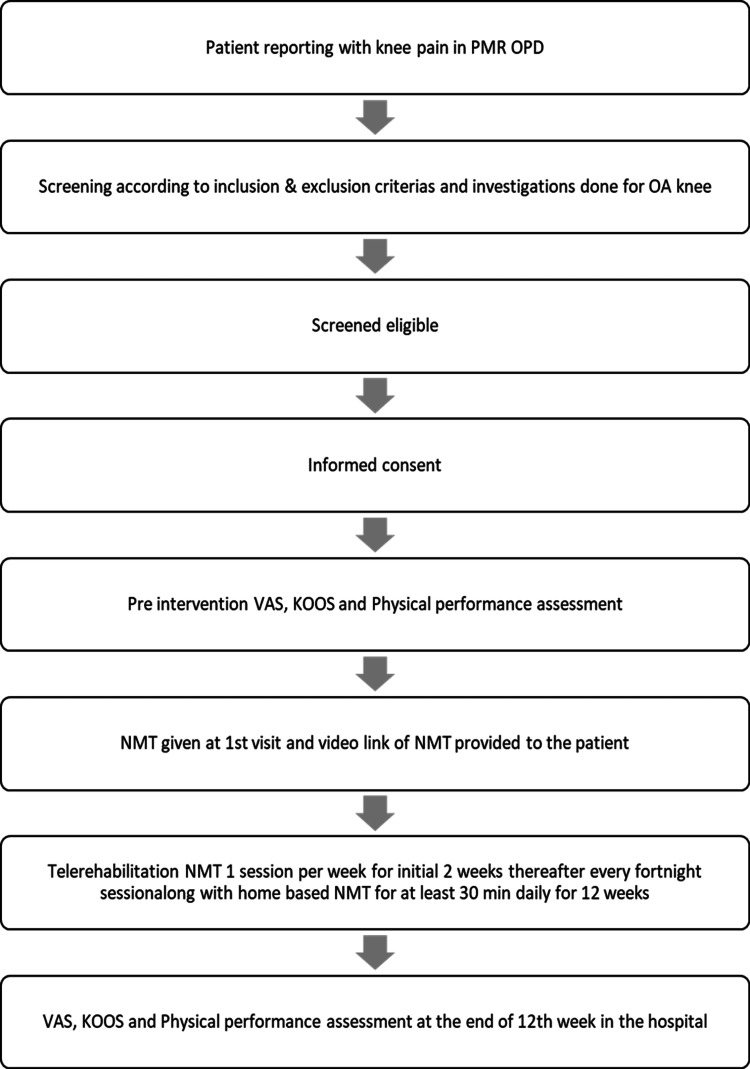
Flowchart of the study PMR: Physical Medicine and Rehabilitation; OPD: Outpatient department; OA: Osteoarthritis; VAS: Visual Analog Scale; KOOS: Knee Injury and Osteoarthritis Outcome Score; NMT: Neuromuscular training

After the final assessment, data analysis was done on 30 patients (Figure 1). 

Intervention

**Figure 2:**
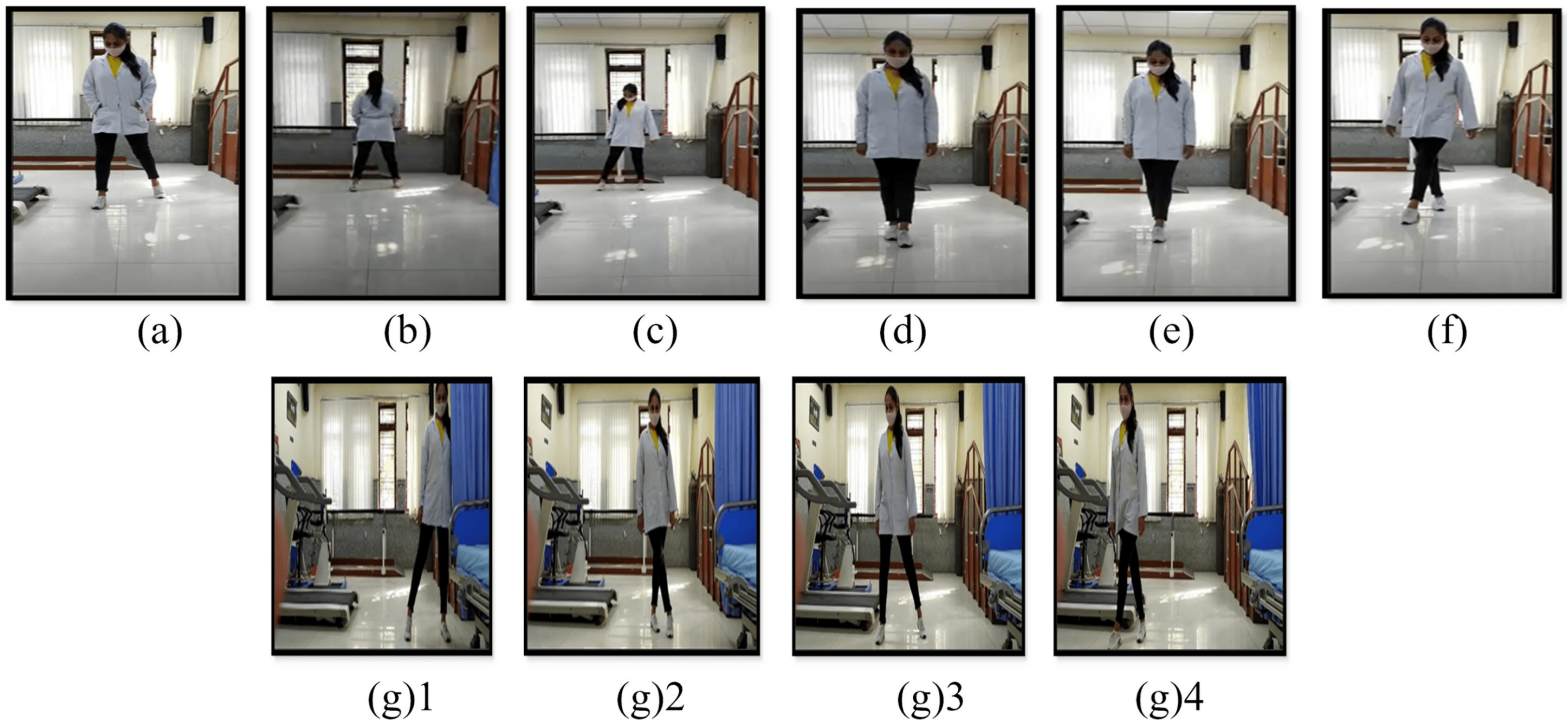
Healthcare worker demonstrating various NMT exercises (a): Wedding march (b): Backward wedding march (c): Side stepping (d): Semi-tandem walk (e): Tandem walk (f): Cross-over walk (g)1-4: Modified grapevine NMT: Neuromuscular training

**Figure 3:**
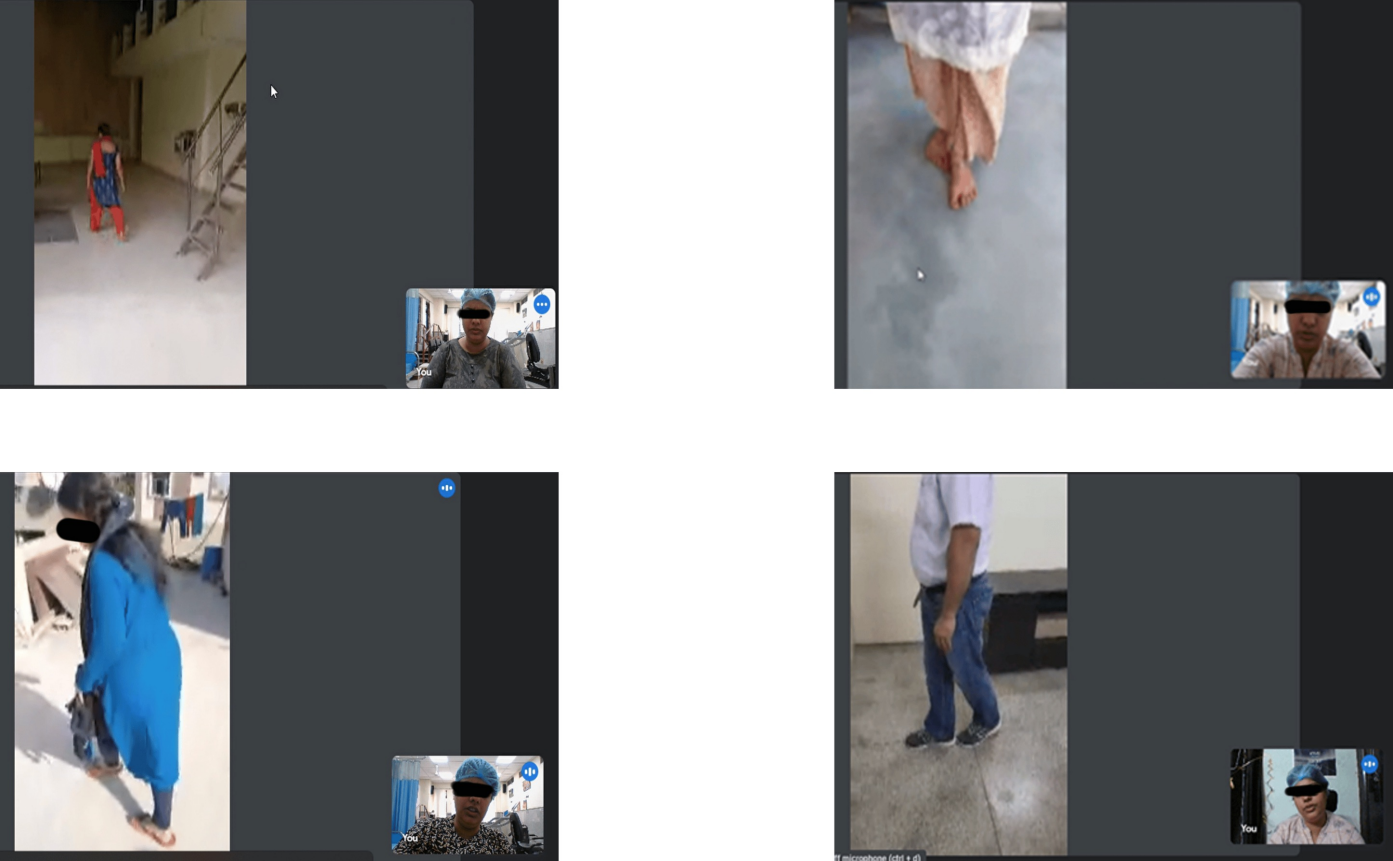
Video snippets of telerehabilitation sessions showing the patient on the left and the doctor shown in the bottom right corner Informed consent was taken from individual patients for the recording and use of the video snippets of the telerehabilitation sessions.

For the initial two weeks, two repetitions of each NMT exercise and third week onward, five repetitions of the exercises were done and recorded as to how many repetitions the patient was able to perform (feedback exercise diary was maintained by the patient) (Table 1, Figures 2, 3) [23].

**Table 1:** NMT exercises NMT: Neuromuscular training

EXERCISE	DESCRIPTION
Wedding march	Step forward and slightly to one side with the leading foot, bring the trailing foot together with the leading foot; alternate the leading foot.
Backward wedding march	As above, stepping backward.
Side stepping	Stand with feet together, step to the side with the leading foot and bring trailing foot back to the leading foot; repeat for the prescribed number of steps, then repeat in the opposite direction.
Semi-tandem walk	Walk heel-to-toe with the heel landing just in front of and medial to the great toe of the opposite foot.
Tandem walk	Advanced version of above; heel lands directly in front of the opposite foot.
Cross-over walk	Walk forward bringing each foot across the midline of the body.
Modified grapevine	Step to the side with the right foot, bring the left foot behind the right, step to the side with the right, bring the left in front of the right; repeat for the prescribed number of steps; change the leading foot and repeat in opposite direction.

Assessment

Each patient was assessed at entry level and at the end of 12th week post TRM of NMT using predefined assessment tools.

Tools for Assessment

For assessing clinical improvement: VAS is used to evaluate the intensity of subjective pain. The patient scores the pain on a scale of 0-100mm (0 mm = no pain, 100 mm = most severe pain) [24]. KOOS's five patient-relevant dimensions are scored separately: pain (nine items); symptoms (seven items); ADL function (17 items); sport and recreation function (five items); and quality of life (four items). A Likert scale is used, and all items have five possible answer options scored from 0 (no problems) to 4 (extreme problems). Each of the five scores is calculated as the sum of the items included. Scores are transformed to a 0-100 scale, with 0 representing extreme knee problems and 100 representing no knee problems [25]. The Hindi version of KOOS is also available and was used where patients were comfortable in Hindi [26]. 

For assessing physical performance: The number of complete chair stands (up and down = one stand) completed in 30 seconds are counted. Higher scores indicate greater physical function. The total time taken to walk 4 × 10 m quickly but safely, excluding turns, is expressed as speed in m/s. Higher walking speeds indicate greater physical function. The total time taken to ascend and descend a flight of nine stairs as quickly and safely as possible is measured. Use of one handrail is permitted if required. Shorter times to complete the test indicate greater physical function [27].

Statistical analysis

The presentation of the categorical variables was done in the form of numbers and percentage (%). On the other hand, the quantitative data were presented as the means ± SD and as median with 25th and 75th percentiles (interquartile range). The data normality was checked by using the Kolmogorov-Smirnov test. In the cases in which the data was not normal, we used nonparametric tests. The comparison of the variables which were quantitative and not normally distributed in nature was analyzed using Wilcoxon signed rank test and variables that were quantitative and normally distributed in nature were analyzed using paired t test across follow-up.

The data entry was done in the Microsoft EXCEL spreadsheet and the final analysis was done with the use of Statistical Package for Social Sciences (SPSS) version 21.0 (IBM, USA).

For statistical significance, a p-value of less than 0.05 was considered statistically significant. 

## Results

**Figure 4:**
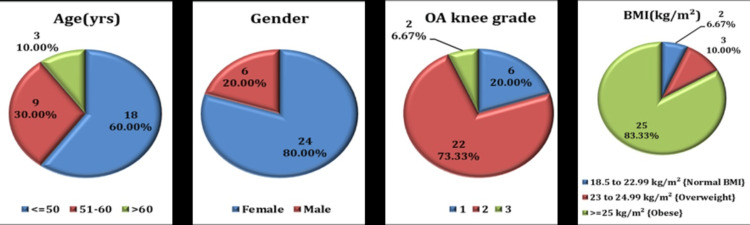
Baseline demographic characteristics of the 30 study participants with OA knee grades 1, 2, 3 OA: Osteoarthritis

In our study, 30 patients with bilateral OA knee were analyzed, in which majority of the cases were in the age group ≤ 50 years, followed by 51-60 years. Females were more affected than males. Most of the cases were of KL grade 2 with the majority of patients having BMI in the obese range (Figure 4). Statistically significant improvement in VAS, KOOS, and physical performance tests at the end of 12 weeks follow up after NMT via telerehabilitation was observed with 95% CI. Mean changes were as follows: VAS -40 (-50 to -40), KOOS +21 (19.62 to 22.38), 30s-CST +3 (2 to 2), 40m-FPWT -0.29 (0.12 to 0.20), 9-Step SCT -3.75 (-6.34 to -3.24). The average set of exercises that the subjects were able to complete is 3 (Table 2).

**Table 2:** Comparison of various outcome measures at baseline and 12 weeks after intervention <0.0001^*^: P value obtained by Wilcoxon signed-rank test; <0.0001^†^: P value obtained by paired t-test VAS: Visual Analog Scale; KOOS; Knee Injury and Osteoarthritis Outcome Score; 30s-CST: 30-second Chair Sit-to-Stand Test; 40m-FPWT: 40-meter Fast-Paced Walk Test; 9-Step SCT: 9-Step Stair-Climb Test

VAS	Mean ± SD	Median (25th-75th percentile)	Range	Median difference (95% CI for median)	P value
At 0 week	62 ± 10.31	60 (52.5-70)	40-80	-40 (-50 to -40)	<0.0001^*^
At 12 weeks	19 ± 7.59	20 (10-20)	10-30		
KOOS knee survey (% symptoms)					
At 0 week	64.57 ± 12.2	64 (61-74)	32-86	21 (14 to 21)	<0.0001^*^
At 12 weeks	83.8 ± 9.17	86 (82-89)	64-96		
KOOS knee survey (% pain)					
At 0 week	61.3 ± 10.95	64(58-68.5)	33-83	25 (20 to 25)	<0.0001^*^
At 12 weeks	86.5 ± 8.13	89(83-92)	69-100		
KOOS knee survey (% functional, daily living)					
At 0 week	64.73 ± 10.16	64 (60-70.5)	41-85	21.90 (19.456 to 24.344)	<0.0001^†^
At 12 weeks	86.63 ± 6.03	88 (84-91)	74-100		
KOOS knee survey (% function, sports and recreational activities)					
At 0 week	36.83 ± 15.4	32.5 (25-50)	10-70	23.67 (20.98 to 26.35)	<0.0001^†^
At 12 weeks	60.5 ± 11.25	60 (50-65)	45-85		
KOOS knee survey (% quality of life)					
At 0 week	41.7 ± 11.11	44 (38-50)	10-56	18 (7 to 13)	<0.0001^*^
At 12 weeks	56.1 ± 15.58	63 (44-69)	25-88		
KOOS knee survey (% total)					
At 0 week	53.67 ± 9	54.5 (50-59)	34-70	21 (19.62 to 22.38)	<0.0001^†^
At 12 weeks	74.67 ± 7.37	76 (73-79)	57-87		
30s-CST (no. of repetitions completed in 30 seconds)					
At 0 week	10.97 ± 2.4	11 (10-12)	6-16	3 (2 to 2)	<0.0001^*^
At 12 weeks	14.07 ± 2.23	14 (12.25-16)	10-18		
40m-FPWT (speed calculated in m/s)					
At 0 week	1.22 ± 0.3	1.2 (1.08-1.41)	0.61-1.69	-0.29 (0.12 to 0.20)	<0.0001^*^
At 12 weeks	1.49 ± 0.4	1.51 (1.333-1.667)	0.04-2.14		
9-Step SCT (measured in s)					
At 0 week	16.13 ± 6.56	14.37 (11.32-18.41)	7.08-35.12	-3.75 (-6.34 to -3.24)	<0.0001^*^
At 12 weeks	11.77 ± 4.68	10.88 (8.272-12.425)	5.52-22.68		
Average of sets of exercise done					
0-14 days	1.78 ± 0.37	1.9 (1.65-2)	1-2.57	3 (3 to 3)	<0.0001^*^
15-90 days	4.84 ± 0.19	4.8 (4.8-5)	4.13-5		

## Discussion

In times of COVID-19, it was very difficult for patients to access healthcare facilities; teleconsultation was used to bridge the gap. There have been previous studies to assess the efficacy of NMT in patients of OA knee. However, there have been no studies assessing the efficacy of NMT in OA knee via telerehabilitation.

In our study total of 30 patients of OA knee with KL grades 1, 2, and 3 were recruited from PMR outpatient department (OPD) who were given NMT sessions via telerehabilitation and were assessed till 12 weeks.

The sample mainly consisted of middle-aged females and the majority of them were educated. The majority of patients had BMI in the obese range emphasizing the already existing literature that obesity increases the prevalence of OA. The main presenting complaint was pain followed by morning joint stiffness and swelling. The majority of patients had grade 2 OA knee according to KL grading.

One of the most severe and incapacitating characteristics of OA knee is pain. It is intermittent and associated with bearing weight [28,29]. As OA knee aggravates, pain starts appearing at rest and at night. It even interferes with sleep [30]. In our study, statistically significant reduction was observed in VAS pain score from a baseline value of 62 ± 10.31 to 19 ± 7.59 with a median improvement (95% CI) of -40 (-50 to -40) at the end of the 12th week (P value < 0.0001). Even though the intensity of pain in patients was significantly reduced it never came to zero.

KOOS evaluates physical activity in different domains like pain, symptoms, ADLs, sports and recreation, function, and quality of life. These domains were assessed to ascertain the response to the NMT. In our study, statistically significant improvement was observed in the subdomains and overall KOOS.

In the symptoms domain, from a baseline value of 64.57 ± 12.2 to 83.8 ± 9.17, median improvement (95% CI) of 21 (14 to 21) at the end of the 12th week (P value < 0.0001) was observed. In the pain domain, from a baseline value of 61.3 ± 10.95 to 86.5 ± 8.13, median improvement (95% CI) of 25 (20 to 25) at the end of the 12th week (P value < 0.0001) was observed. In functional ADLs domain, from a baseline value of 64.73 ± 10.16 to 86.63 ± 6.03, mean improvement (95% CI) of 21.90 (19.456 to 24.344) at the end of the 12th week (P value < 0.0001) was observed. In the sports and recreational activities domain, from a baseline value of 36.83 ± 15.4 to 60.5 ± 11.25, mean improvement (95% CI) of 23.67 (20.98 to 26.35) at the end of the 12th week (P value < 0.0001) was observed. In the quality-of-life domain, from baseline value of 41.7 ± 11.11 to 56.1 ± 15.58, median improvement (95% CI) of 18 (7 to 13) at the end of 12th week (P value < 0.0001) was observed. In total KOOS, from a baseline value of 53.67 ± 9 to 74.67 ± 7.37 03, mean improvement (95% CI) of 21 (19.62 to 22.38) at the end of the 12th week (P value < 0.0001) was observed.

In our study, statistically significant improvement was observed in the three parameters of physical performance tests. In 30s-CST, from a baseline value of 10.97 ± 2.4 and 14.07 ± 2.23, median improvement (95% CI) of 3 (2 to 2) at the end of 12th week (P value < 0.0001) was observed. In 40m-FPWT, from baseline value of 1.22 ± 0.3 and 1.49 ± 0.4, median improvement (95% CI) of 0.29 (0.12 to 0.20) at the end of 12th week (P value < 0.0001) was observed. In 9-Step SCT, from a baseline value of 16.13 ± 6.56 and 11.77 ± 4.68, median improvement (95% CI) of -3.75 (-6.34 to -3.24) at the end of the 12th week (P value < 0.0001) was observed.

In our study, a statistically significant increase was seen in the average number of sets of exercises performed in 15-90 days as compared to 0-14 days (P value < 0.0001).

In a study conducted by Ageberg et al. on the effects of NMT-total joint replacement on patient-reported outcomes and physical function in severe primary hip or knee OA, statistically significant improvement was seen in KOOS and physical performance tests [16].

In a randomized controlled trial by Bennell et al. on neuromuscular versus quadriceps strengthening exercise in patients with medial knee OA and varus malalignment, both groups showed similar significant reductions in pain and improvement in physical function on VAS and WOMAC after 12 weeks of exercises [31].

In a study conducted by Skou and Roos, evidence-based education and supervised neuromuscular exercises were delivered by certified physiotherapists nationwide [32]. Statistically significant improvement in patients' symptoms and physical function assessed by VAS, KOOS, and physical function tests were observed in our study. There was a reduction in intake of painkillers and sick leave among the patients. The lifestyle changes introduced by education and supervised exercise were largely maintained for one year [32].

In a study conducted by Rashid et al. on comparison of NMT with QT on gait and WOMAC in patients with knee OA and varus malalignment, significant improvement was seen in VAS and WOMAC after 12 weeks of NMT in comparison to QT [22].

The NMT program was aimed to produce controlled movements through coordinated muscle activity and dynamic stability. Previous research has reported reduced functional performance and sensory-motor deficiencies in patients with OA knee [33]. Functional tasks are limited by functional instability in the OA knee [34]. The data from our study suggest that there was an improvement in the performance of knee joints during dynamic activities like walking and in performing ADL.

Relevance and role of telerehabilitation exercise training in the post-pandemic era and its usefulness to improve patient care and compliance can be explained by the benefits of telerehabilitation. These include: cost effectiveness for both the healthcare provider and the patient, as there is no cost of travel for the patients to come to the hospital from remote areas; specialized care delivery to rural areas; increased accessibility, leading to more timely intervention, improved patient outcomes, and reduction in the progression of disabilities; real-time monitoring, prompt feedback, as well as personalized plans delivered to individual patients [35].

Limitation

A larger sample size and control group is warranted for further consolidation of our findings.

## Conclusions

From our study, we found significant improvement in VAS, KOOS, and physical performance outcomes in OA knee patients after NMT via telerehabilitation. We can conclude that NMT in patients with OA knee is a good choice of treatment modality via telerehabilitation. However, a larger sample size and a control group is warranted for further consolidation of our findings.
